# Protozoal food vacuoles enhance transformation in *Vibrio cholerae* through SOS-regulated DNA integration

**DOI:** 10.1038/s41396-022-01249-0

**Published:** 2022-05-16

**Authors:** Md Hafizur Rahman, Khandaker Rayhan Mahbub, Gustavo Espinoza-Vergara, Angus Ritchie, M. Mozammel Hoque, Parisa Noorian, Louise Cole, Diane McDougald, Maurizio Labbate

**Affiliations:** 1 School of Life Sciences, Faculty of Science, University of Technology Sydney, Sydney, NSW Australia; 2 South Australian Research and Development Institute, Urrbrae, SA Australia; 3 Australian Institute for Microbiology & Infection, University of Technology Sydney, Sydney, NSW Australia

**Keywords:** Bacteriology, Microbial genetics

## Abstract

*Vibrio cholerae*, the bacterial pathogen responsible for the diarrheal disease cholera, resides in the aquatic environment between outbreaks. For bacteria, genetic variation by lateral gene transfer (LGT) is important for survival and adaptation. In the aquatic environment, *V. cholerae* is predominantly found in biofilms associated with chitinous organisms or with chitin “rain”. Chitin induces competency in *V. cholerae*, which can lead to LGT. In the environment, *V. cholerae* is also subjected to predation pressure by protist. Here we investigated whether protozoal predation affected LGT using the integron as a model. Integrons facilitate the integration of mobile DNA (gene cassettes) into the bacterial chromosome. We report that protozoal predation enhances transformation of a gene cassette by as much as 405-fold. We show that oxidative radicals produced in the protozoal phagosome induces the universal SOS response, which in turn upregulates the integron-integrase, the recombinase that facilitates cassette integration. Additionally, we show that during predation, *V. cholerae* requires the type VI secretion system to acquire the gene cassette from *Escherichia coli*. These results show that protozoal predation enhances LGT thus producing genetic variants that may have increased capacity to survive grazing. Additionally, the conditions in the food vacuole may make it a “hot spot” for LGT by accumulating diverse bacteria and inducing the SOS response helping drive genetic diversification and evolution.

## Introduction

*Vibrio cholerae* inhabits coastal and estuarine water environments and is the etiological agent of the often-fatal diarrheal disease, cholera [1]. In the environment, *V. cholerae* is faced with numerous pressures including interactions with free-living heterotrophic protists or protozoa [2]. Protozoa package bacteria into food vacuoles (phagosomes) that become acidified and filled with toxic components that aid digestion, including oxidative radicals. Despite this hostile environment, *V. cholerae* has evolved mechanisms to resist protozoal predation or manipulate the digestive process in order to create a protective niche [3]. In *Acanthamoeba castellanii, V. cholerae* can escape digestion and be exocytosed to the environment or shuttled internally to the contractile vacuole where it replicates, remaining during encystment before escaping [4]. In *A. castellanii* and *Tetrahymena pyriformis*, food vacuoles containing *V. cholerae* are expelled to the environment, where they provide a protective habitat and enhance survival and infectious potential [5].

*V. cholerae* biofilm formation on chitin surfaces provides protection, a nutritive substrate [1] and induces natural competence facilitating lateral gene transfer (LGT) through transformation [6]. Growth on chitin also induces the type VI secretion system (T6SS), resulting in lysis of neighboring cells and subsequent release of DNA [7] that can be taken up for transformation. In addition to DNA uptake, DNA transformation requires integration into a replicon such as the chromosome before it can be expressed and passed onto progeny [8]. *V. cholerae* pandemic strains have been heavily influenced by LGT [9, 10] as the two main virulence factors, cholera toxin (CTX) that is responsible for diarrhea and an intestinal adhesin and the toxin co-regulated pilus (TCP) are present on mobile genetic elements (MGEs) [11, 12]. MGEs are important in facilitating the integrative step of LGT, particularly where DNA cannot integrate through RecA-mediated homologous recombination. The integron in *V. cholerae* is a site-specific recombination system that integrates circular MGEs. The integron-integrase is induced by the SOS response [13], a regulatory cascade in bacteria that is induced by the DNA repair protein, RecA, which accumulates around single-stranded DNA (ssDNA) arising from processes such as stalled chromosome replication or LGT and activates its cleavage of the repressor, LexA [14]. Primarily, SOS induces DNA repair genes, however, it also induces site-specific recombinases in MGEs, such as prophages and genomic islands, linking DNA transfer with DNA integration [15, 16].

Here, for the first time, we show that the integron-integrase is expressed in the food vacuoles of protozoa and that this expression is due to oxidative radicals and induction of the SOS response. The induction of the SOS response enhances transformation of a gene cassette by as much as 405-fold by upregulating integron-integrase expression and gene cassette integration. In our assays, the gene cassette was transferred as free DNA or from within an *Escherichia coli* host, but only when T6SS was functional. Taken together, our results show that protozoal predation enhances genetic diversification that may assist adaptation to grazing pressure and contribute to the evolution of *V. cholerae* and possibly, to other aquatic bacteria.

## Materials and methods

### Bacterial strains, plasmids, oligonucleotides, and growth conditions

Bacterial strains, plasmids and primers used in this study are listed in Supplementary Tables 1, 2. Bacterial strains were routinely grown at 37 °C in Luria Bertani (LB) medium. For *V. cholerae*, kanamycin (Kan), chloramphenicol (Cm) and spectinomycin (Spc) were used at 50, 5, and 100 µg ml^−1^, respectively. For *E. coli* WM3064, diaminopimelic acid (DAP) was supplemented to a final concentration of 0.3 mM and Cm and Kan were used at 25 and 50 µg ml^−1^, respectively. To induce gene expression in strains carrying pSU-pBAD, a final concentration of 0.2% (w/v) L-arabinose was added to the growth medium. Plasmid DNA was extracted using the PureYield Plasmid Miniprep Systems kit (Promega, Wisconsin, USA). PCR fragments were purified using the ISOLATE II PCR and Gel Kit (Bioline, London, England). Oligonucleotides were obtained from Integrated DNA Technologies (Singapore).

### Growth and maintenance of protozoa

*T. pyriformis* was routinely cultured in 10 mL of peptone yeast-glucose (PYG) medium in 25 cm^2^ tissue culture flasks with ventilated caps (Sarstedt Inc., Numbrecht, Germany) and incubated statically at 23 °C. PYG medium consists of 20 g l^−1^ proteose peptone, 1 g l^−1^ yeast extract and 0.1 M glucose dissolved in 1 L of 0.1 × M9 Salts Solution (1.28 g L^−1^ Na_2_HPO_4_, 0.3 g L^−1^ KH_2_PO_4_, 0.05 g L^−1^ NaCl, 0.1 g L^−1^ NH_4_Cl). Prior to experiments, 500 µl of *T. pyriformis* was passaged in 20 mL of 0.35 × Nine Salts Solution (NSS; 6.16 g L^−1^ NaCl, 0.515 g L^−1^ Na_2_SO_4_, 0.028 g L^−1^ NaHCO_3_, 0.0875 g L^−1^ KCl, 0.014 g L^−1^ KBr, 0.655 g L^−1^ MgCl_2_.6H_2_O, 0.1435 g L^−1^ CaCl_2_.2H_2_O, 0.0028 g L^−1^ SrCl_2_.6H_2_O and 0.0028 g L^−1^ H_3_BO_3_) [17] and incubated overnight statically at 23 °C to allow the culture to adapt to the nutrient free medium. *A. castellanii* was routinely cultured and passaged as above with the exception of incubation at 30 °C. Before use in experiments, *A. castellanii* was passaged for 3 d prior to obtain freshly growing cells, washed with 0.1 × M9 Salts Solution and resuspended in 0.1 × M9 Salts Solution supplemented with 1% glucose. Protozoa were enumerated microscopically using a hemocytometer and numbers were adjusted as described below.

### Construction of integron-integrase reporter strain, artificial gene cassette, and mutants

The *V. cholerae* integron-integrase reporter was created by chitin transformation of p4640 [18], a suicide vector containing an *intIA*::*gfp* transcriptional fusion, into *V. cholerae* A1552. *V. cholerae* mutants were created by using splicing overlap extension PCR to construct mutated alleles followed by natural transformation [19]. The FRT-*cat*-FRT cassette interrupting the Δ*intIA* mutant allele used for selection was excised via FLP-mediated recombination as previously described [20]. The *lexA*(ind^−^) mutant was constructed by cloning *lexA* into pSW4426T, a suicide vector containing an arabinose-inducible *ccdB* counter-selectable marker and introducing an A91D allele by PCR. Transformations of *ccdB* constructs were conducted using the CcdB-resistant *E. coli* ∏3813 strain (Supplementary Table 1). The plasmid containing the mutated *lexA*(ind^−^) allele was chitin transformed into A1552 and a recombinant obtained on selective medium. Counter-selection was carried out on non-selective medium containing 0.2% arabinose and colonies screened for the *lexA*(ind^−^) allele using amplification refractory mutation system (ARMS) PCR. To investigate gene cassette transfer into the *V. cholerae* integron, a circular gene cassette that doubles as a plasmid with a conditional origin of replication (oriR6K), pKC01, was constructed. pKC01 was constructed via amplification of a gene cassette (locus tag RS15635 in *V. cholerae* N16961, NZ_CP028828) with TnFGL3 inserted [21] that was then circularized by ligation. pKC01 contains a kanamycin resistance gene (*nptII*), promoterless *gfpmut3* and *lacZ* and a single *V. cholerae*-specific *attC* site with *gfpmut3-lacZ-*oriR6K*-nptII* downstream of the GTTRRRY *attC* recombination site (see Supplementary Fig. 1 for a schematic of pKC01).

### Co-incubation of bacteria with protozoa

Co-incubation assays were conducted in 24-well microtiter plates containing 1 mL of co-culture per well (Nunclon Delta surface, ThermoFisher Scientific, Denmark), as previously described [5, 22]. Briefly, *T. pyriformis* and *A. castellanii* were enumerated and adjusted to 10^3^ and 10^4^ cells ml^−1^, respectively. For *A. castellanii*, plates were incubated at 23 °C for 1 h to facilitate attachment to the plate surface. Bacterial cells (strains of *V. cholerae* or *E. coli)* were added at a final concentration of 10^8^ cells ml^−1^ for co-incubation with *T. pyriformis* and 10^7^ cells ml^−1^ for co-incubation with *A. castellanii*, and subsequently incubated statically at 23 °C for 4 h.

In LGT co-incubation experiments using pKC01, chitin-competent *V. cholerae* cells were prepared using commercially available chitin flakes as previously described [19]. Briefly, cells were grown at 30 °C in 10 mL LB to an OD_600_ of ~0.4 before centrifugation and washing, and then resuspended in 1 mL of 0.7% Sea Salts solution (Sigma Aldrich). To a tube containing 900 μl of sterile 0.7% Sea Salts solution and 50–70 mg chitin flakes, 100 μl of the cell suspension was added and allowed to incubate statically overnight. Subsequently, 500 µl of supernatant was carefully aspirated and discarded and 500 µl of fresh 0.7% Sea Salts solution was added before the tube was rigorously vortexed for 5 min to detach the chitin-competent *V. cholerae* cells from the chitin flakes. The chitin flakes were left to settle for 2–3 min before the supernatant was carefully aspirated. From multiple tubes, ~5 mL of supernatant was collected, centrifuged and adjusted to an OD_600_ of 1.0 (equivalent to 10^9^ cells ml^−1^). pKC01 was provided as both free DNA (5 µg) and as a plasmid in *E. coli* WM3064 in co-cultures of *V. cholerae* A1552 and protozoa.

Transformants were extracted from the protozoa for selective plating. For surface-attached *A. castellanii*, the co-culture was carefully washed three times with sterile 0.1 × M9 containing 1% glucose before an equal volume of the same culture media containing 1% Triton-X was added to lyse the amoeba and release the internalized bacterial cells. In the case of *T. pyriformis*, co-cultures were aspirated from the plate and gently collected by centrifugation at 400 × *g* for 10 min before the supernatant was carefully aspirated and discarded. The pellet containing *T. pyriformis* cells was then gently washed 3 times with sterile 0.35 × NSS and resuspended in an equal volume of 0.35 × NSS containing 1% Triton-X to lyse the protozoa. Appropriate dilutions of lysates were plated on agar plates containing Kan to select for transformants. Transformation frequencies were normalized by dividing the number of Kan resistant CFU by the total number of intracellular CFU [19]. For each transformation, ten random colonies were selected and confirmed to contain the *V. cholerae ompW* and *nptII* genes by PCR (Supplementary Table 2).

### Microscopic imaging

For confocal laser scanning microscopy (CLSM) of co-cultures containing *T. pyriformis* and *V. cholerae*, the samples were collected after 4 h co-incubation: 200 µl of co-culture was stained with the red fluorescent fixable lipophilic dye FM4-64 FX (Invitrogen, Carlsbad CA) according to the manufacturer’s instructions, collected by centrifugation (400 × *g*, 10 min) and fixed by resuspending in phosphate buffered saline (PBS) containing 2% paraformaldehyde (pH 7.2–7.4) for 10 min at room temperature. Fixed cells were washed three times by centrifugation (400 × *g*, 10 min) and resuspended in PBS before staining with DAPI (diamidino-2-phenylindole, ThermoFisher Scientific, USA) according to the manufacturer’s instructions. After staining, cells were washed three times as above and resuspended in PBS before mounting on microscope slides; 10 μl of fixed sample was placed on a glass slide and allowed to dry at room temperature for 15 min. After drying, 10 μl of Vectashield HardSet Antifade Mounting Medium (Vector Laboratories, USA) was added and the sample sealed with a glass coverslip (1.5 mm thickness, Neuvitro, USA). Samples were placed in the dark at RT overnight to allow the mounting media to set and imaged the next day or stored at 4 °C for later observation. Mounted samples were imaged using an inverted Nikon A1 confocal laser scanning microscope using 405, 488, and 561 nm excitation to image DAPI, GFP and FM4-64 fluorescence, respectively. Single and Z-stack multi-channel fluorescence images of samples were collected, and corresponding transmitted light (TL) images were obtained using the TL detector. The co-culture containing *A. castellanii* and *V. cholerae* were imaged live with wide-field fluorescence microscopy with samples placed in CellCarrier 96—Ultra Microplates (PerkinElmer, UK) after 4 h of co-incubation. All images were analyzed using FIJI [23] and Imaris v9.2 (Bitplane, USA) analysis software.

### Quantification of GFP fluorescence

Following co-incubation*, T. pyriformis* cells were collected by centrifugation (400 × *g* for 10 min), the supernatant was aspirated, and the pellet gently washed 3 times with sterile 0.35 × NSS. The *T. pyriformis* was lysed by resuspending in an equal volume of 0.35 × NSS containing 1% Triton-X. For the surface-attached *A. castellanii*, cells were gently washed three times with sterile 0.1 × M9 and resuspended in an equal volume of 0.1 × M9 containing 1% Triton-X. GFP fluorescence was quantified as previously described [24]. Briefly, 200 µl of sample was transferred into a 96-well microtiter plate (Corning^®^ 96-Well Black Polystyrene Microplate flat bottom clear), GFP fluorescence measured at 488 nm excitation and 530 nm emission wavelengths and optical density at 600 nm were determined using a multimode microplate reader (Spark microplate reader, TECAN, Switzerland). Background fluorescence was determined using cultures containing the wild-type *V. cholerae* strain. GFP fluorescence was normalized to the cell density of bacterial cells and presented using the following formula:

$${{{{{\rm{Relative}}}}}}\; {{{{{\rm{fluorescence}}}}}}\; {{{{{\rm{units}}}}}}\;=	 \, [{{{{{\rm{Reporter}}}}}}\; {{{{{\rm{strain}}}}}}\; {{{{{\rm{fluorescence}}}}}}\; {{{{{\rm{intensity}}}}}}/{{{{{\rm{cell}}}}}}\; {{{{{\rm{density}}}}}}\; ({{{{{\rm{OD}}}}}}_{600})]\\ 	- [{{{{{\rm{Background}}}}}}\; {{{{{\rm{fluorescence}}}}}}\; {{{{{\rm{intensity}}}}}}/{{{{{\rm{cell}}}}}}\; {{{{{\rm{density}}}}}}\; ({{{{{\rm{OD}}}}}}_{600})]/1000$$

### Quantification and quenching of oxidative radicals

Following a 4 h co-incubation with wild-type *V. cholerae*, Triton-X (1% final concentration) was added to the protozoan cells and the sample was centrifuged at 20,598 × *g* for 2 min to collect the supernatant containing the oxidative radicals. Oxidative radicals were measured using the dye 2′,7′-dichlorofluorescin diacetate (DCF-DA) as previously described [25]. Briefly, 10 µM DCF-DA (Sigma, USA) was added and the supernatant incubated at 37 °C for 30 min. The samples (200 µl) were transferred to 96-well microtiter plates (Corning 96-Well Black Polystyrene Microplate flat bottom clear) and fluorescence was measured using a multimode microplate reader (Spark microplate reader, TECAN, Switzerland) with 485 nm excitation and 525 nm emission wavelengths. For quenching experiments, *T. pyriformis* and *A. castellanii* cells were treated with 100 mM of the quencher, thiourea (pH 7.0; Sigma, USA) [26], before addition of bacterial cells. Fluorescence data from the reader was divided by 1000 before plotting on a graph.

### Quantitative reverse-transcriptase PCR (qRT-PCR)

Total RNA was prepared from co-incubation assays of *V. cholerae* with *T. pyriformis* and *A. castellanii* in 24-well plates. Bacterial cells from within protozoa were collected as described above and RNA was extracted by lysozyme digestion followed by use of the Aurum Total RNA mini kit (Bio-Rad, Hercules, CA, USA) according to the manufacturer’s instructions. The concentration of RNA was measured by spectrophotometry (NanoDrop ND-1000; NanoDrop Technologies). Complementary DNA (cDNA) was prepared from 400 ng RNA from each sample by iScript Reverse Transcription (Bio-Rad, Hercules, CA, USA) following the manufacturer’s instructions. Quantitative reverse-transcriptase PCR (qRT-PCR) was conducted using SsoAdvanced Universal SYBR Green Master Mix (Bio-Rad, Hercules, CA, USA) and a QuantStudio 6 Flex Real-Time PCR System using *V. cholerae* integron-integrase specific primers (intIA-1F/intIA-1R) (Supplementary Table 2). Transcription of *intIA* was determined relative to the transcription of *gyrA (*gyrA_F/gyrA_R*)* (Supplementary Table 2) using the comparative Ct (ΔΔCt) method.

### Statistical analysis

All statistical analyses were performed using GraphPad Prism version 7.01 for Windows, GraphPad Software, La Jolla California, USA (www.graphpad.com). Two-tailed student’s *t* tests were used to compare means between experimental samples and controls. For experiments with multiple samples, one-way ANOVAs were used with Tukey’s or Dunnett’s Multiple Comparison Test post hoc comparisons of means.

## Results

### The *V. cholerae* integron-integrase is induced in protozoal phagosomes

**Fig. 1:**
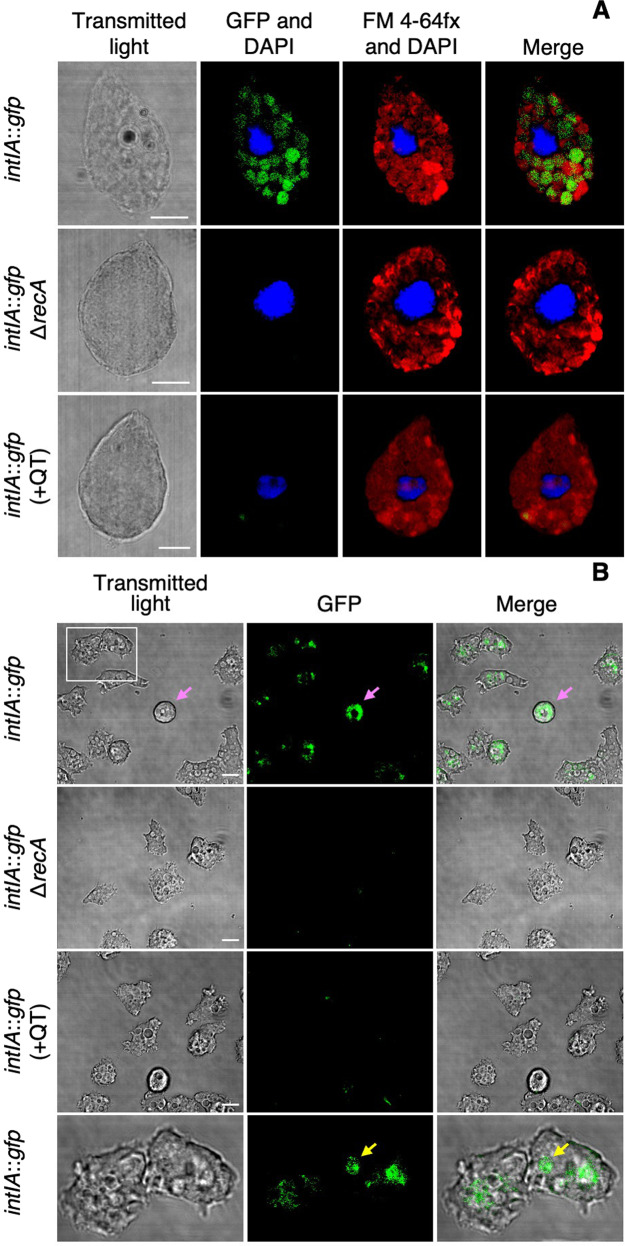
Microscopy of coincubations of *V. cholerae* strains with, *T. pyriformis* or *A. castellanii*. The *V. cholerae* integron-integrase reporter strain (*intIA*::*gfp*) contains a transcriptional fusion of *intIA* and *gfp*. The *intIA*::*gfp*,Δ*recA* strain is the reporter strain with *recA* deleted. +QT indicates the addition of 100 mM of the quencher, thiourea. **A** Images show *T. pyriformis* after feeding on *V. cholerae* using transmitted light and, composite images from confocal microscopy showing GFP fluorescence (green) and DAPI stain fluorescence (blue) of *V. cholerae* and the protozoan nucleus, respectively; red (FM4-64FX) for lipophylic membranes and DAPI; and merged fluorescence channels (GFP + FM4-64FX + DAPI) respectively. **B** Images of amoebae after co-incubation with *V. cholerae* imaged with transmitted light, wide-field fluorescence microscopy showing GFP fluoresecence and, merged transmission and fluoresecence channels. Pink arrow indicates an ameobal cyst. Lower panels are an inset of the white box indicated in top row panel with the yellow arrow indicating a food vacuole. Scale bar 10 µM.

To determine whether the *V. cholerae* integron-integrase is induced during co-incubation with protozoa, a reporter strain containing a transcriptional fusion of *intIA* with *gfp* was co-incubated with the suspension-feeding *T. pyriformis* or the surface-feeding *A. castellanii*. Following 4 h of co-incubation, confocal microscopic imaging revealed the presence of GFP-expressing *V. cholerae* cells in the phagosome of both protozoa (Fig. 1, Supplementary Video 1, Supplementary Fig. 2). In addition, GFP fluorescent cells were observed in cysts (Fig. 1B, pink arrow) of *A. castellanii. V. cholerae* cells are known to transit to the contractile vacuole from the food vacuole [4] before encystation.

**Fig. 2:**
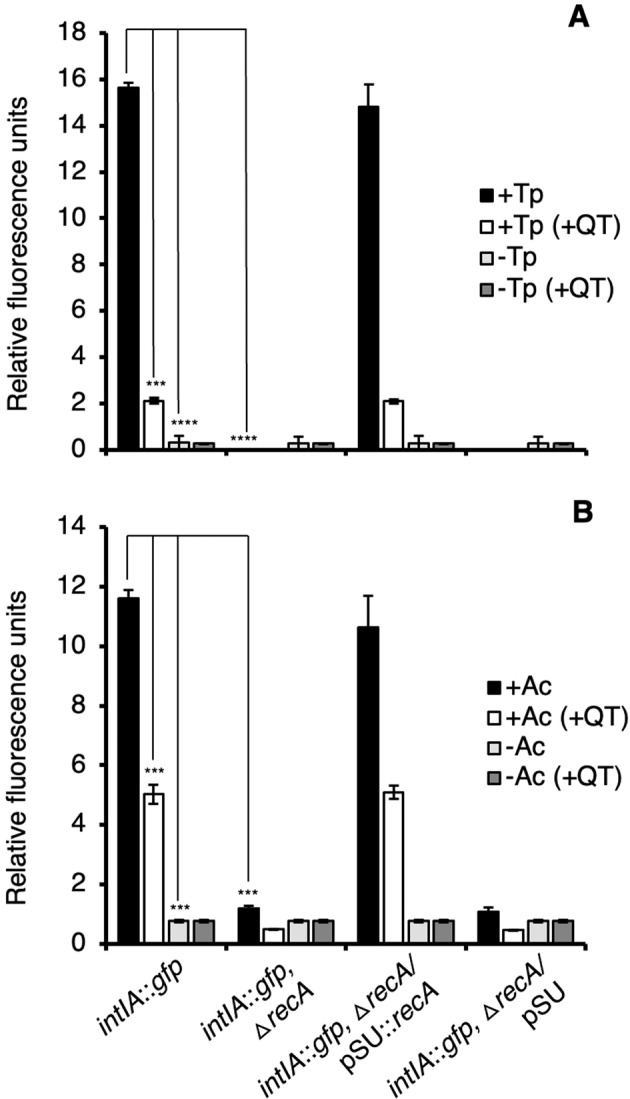
GFP fluorescence of internalized *V. cholerae* integron-integrase reporters strains recovered from protozoa. GFP fluorescence from *V. cholerae* strains recovered from within *T. pyriformis* (+Tp) (**A**) and *A. castellanii* (+Ac) (**B**) and from media controls (−Tp and −Ac). The *V. cholerae intIA*::*gfp* reporter strain contains a transcriptional fusion of *intIA* with *gfp*. The *intIA*::*gfp*,Δ*recA* is the reporter strain with *recA* deleted and the *recA*-complemented strain (*intIA*::*gfp*,Δ*recA*/pSU::*recA*) and vector control strain (*intIA*::*gfp*,Δ*recA*/pSU). GFP fluorescence of internalized bacterial cells recovered from purified protozoa was measured after 4 h incubation. +QT indicates the addition of 100 mM of the quencher, thiourea. Error bars represent the standard deviations of three independent experiments. Significance was calculated using one-way ANOVA and Tukey’s multiple comparisons test (***p < 0.0001; ****p < 0.00001).

**Fig. 3:**
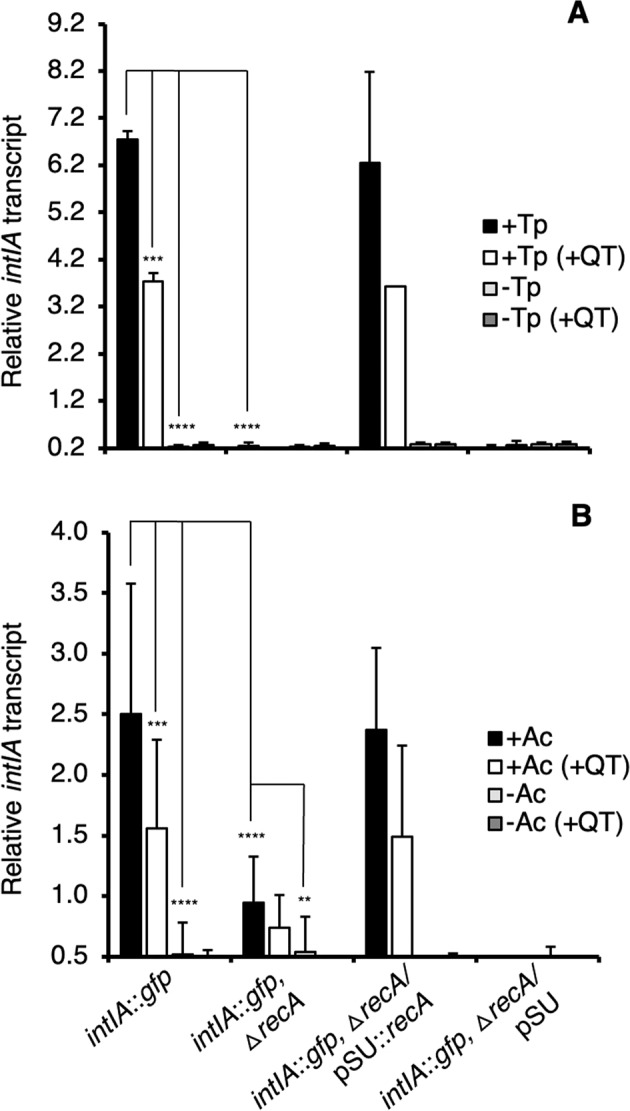
qRT-PCR of *intIA* of internalized *V. cholerae* strains following 4 h of co-incubation with protozoa. *intIA* transcript from *V. cholerae*strains from within *T. pyriformis* (+Tp) (**A**) and *A. castellanii* (+Ac) (**B**) and from media controls (−Tp and −Ac). *intIA* transcript was quantified in wild-type (WT), Δ*recA* and *recA*-complemented strain (Δ*recA*/pSU::*recA*) and vector control strain (Δ*recA*/pSU). Cells external to the protozoa were removed and total RNA was extracted from purified protozoa. +QT indicates the addition of 100 mM of the quencher, thiourea, to the co-incubation. *intIA* transcription levels were normalized to *gyrA* by using the comparative Ct (ΔΔCt) method. Error bars represent the standard deviations of three independent experiments. Significance was calculated using one-way ANOVA and Tukey’s multiple comparisons test (**p < 0.001; ***p < 0.0001; ****p < 0.00001).

To quantify the level of integron-integrase induction, the intensity of the GFP fluorescence was measured using a spectrofluorometer after 4 h co-incubation. Compared to the no protozoa control, GFP fluorescence was 53-fold and 15-fold higher when extracted from within *T. pyriformis* and *A. castellanii*, respectively (Fig. 2). To confirm *intIA* induction occurred in the protozoa, qRT-PCR of RNA extracted from purified protozoa was performed and results showed that after 4 h, the relative expression of *intIA* in the wild type was 29-fold and 4.8-fold higher in *T. pyriformis* and *A. castellanii*, respectively, than in the controls (Fig. 3).

### Gene cassette transformation is enhanced in intracellular *V. cholerae* and requires T6SS when the cassette is provided in another bacterial host

**Fig. 4:**
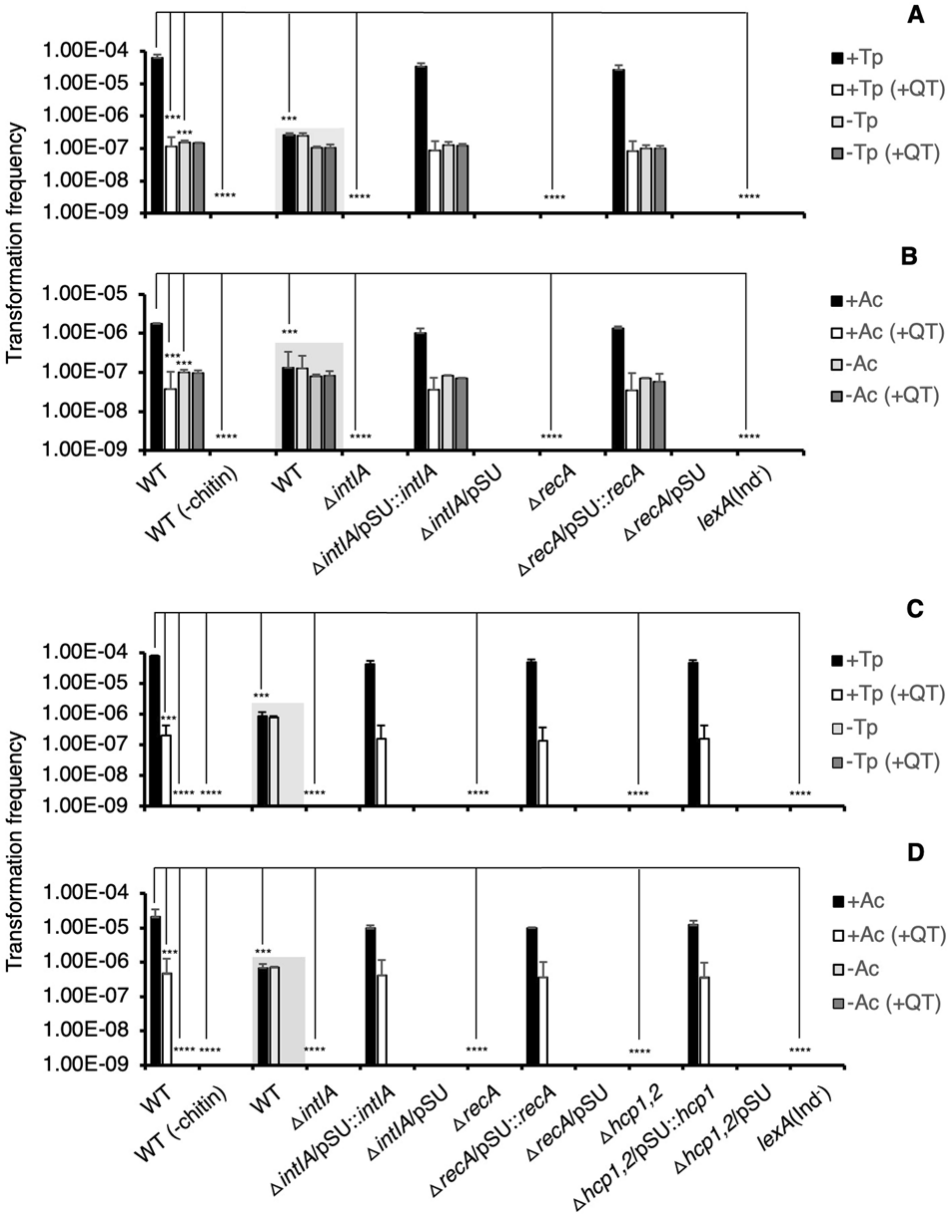
Frequency of transformation in *V. cholerae* strains co-incubated with *T. pyriformis* (Tp) and *A. castellanii* (Ac). Transformation assays were assessed in the wild-type (WT), the Δi*ntIA*, Δ*recA*, Δ*hcp1,2, lexA*(Ind^-^) mutants and the *intIA*-complemented (Δ*intIA*/pSU::*intIA*), *recA*-complemented (Δ*recA*/pSU::*recA*) and *hcp1*-complemented (Δ*hcp1,2*/pSU-pBAD::*hcp1*) strains and vector control strains (Δ*recA*/pSU, Δ*intIA*/pSU and Δ*hcp1,2*/pSU). In coincubations with protozoa, *V. cholerae* must be pre-grown on chitin for successful transfer of DNA. pKC01 and pCVD442::Δ*rtx* (bar data in gray box) were provided as either free DNA (**A**, **B**) or carried within *E. coli* WM3064 (**C**, **D**). +QT indicates the addition of 100 mM of the quencher, thiourea, to the co-incubation. Error bars represent the standard deviations of three independent experiments. Significance was calculated using one-way ANOVA and Tukey’s multiple comparisons test (***p < 0.0001; ****p < 0.00001). Where no data is provided for strains and treatments is non-detectable transformation.

Given that the *V. cholerae* integrase is induced in the phagosomes of *T. pyriformis* and *A. castellanii*, an assay was conducted to determine if gene cassette transformation was enhanced. The artificial gene cassette, pKC01 (5 µg), was provided as purified DNA in co-incubation of *V. cholerae* and protozoa as well as in the no protozoa controls. Only *V. cholerae* wild-type cells that were pre-grown on chitin produced pKC01 transformants in both the protozoal treatments and controls (Fig. 4A, B). In the presence of *T. pyriformis* and *A. castellanii*, transformation efficiency was 405- and 18-fold higher than for chitin transformation in the absence of protists, respectively (Fig. 4A, B). No transformants were obtained using the integron-integrase mutant (Δ*intIA*) and gene complementation restored the phenotype (Fig. 4A, B). PCR spanning the *attI* region (using primers IntIAF-2 and VCH3-R) on 10 pKC01 transformants resulted in 4 giving sizes consistent with insertion of pKC01 at *attI* (~7200-bp), 4 consistent with no insertion at *attI* (~600-bp) and one amplicon of a larger size (~1500-bp) than the wild type with sequencing determining that an existing *V. cholerae* gene cassette had relocated into *attI* (sequence provided in Supplementary Information). Of the four transformants showing no insertion at *attI*, PCR using primers that bind across the array showed insertion of pKC01 at a site(s) between the 26th and 38th cassettes relative to *attI*. A suicide plasmid (pCVD442::Δ*rtx*) that can only integrate by homologous recombination did not show enhanced transformation in the presence of protozoa and gave similar efficiencies to pKC01 in the absence of protozoa (Fig. 4A, B) confirming that enhanced pKC01 transformation is due to enhanced integron-integrase expression.

To determine whether protozoal predation enhances transformation of pKC01 contained within a neighboring bacterial cell, *E. coli* WM3064 carrying pKC01 was used as a donor in the gene cassette transformation assay. *V. cholerae* cells pre-grown on chitin were capable of uptake and integration of pKC01 from *E. coli* WM3064 at a transformation frequency of 7.73 × 10^−5^ and 2.06 × 10^−5^ in *T. pyriformis* and *A. castellanii*, respectively (Fig. 4C, D). Transformation of pKC01 was not detected in the no protozoa controls, in the absence of pre-growth on chitin or in the Δ*intIA* mutant. Complementation of the Δ*intIA* mutant restored transformation back to wild-type levels (Fig. 4C, D). To determine whether the T6SS is required for transformation of pKC01 from *E. coli* WM3064, a double *hcp* mutant incapable of producing T6SS (Δ*hcp1,2*) was tested in transformation assays. No transformants were detected in either the protozoan treatments or in the no protozoa controls when chitin-grown *V. cholerae* was used (Fig. 4C, D). In contrast, the levels of transformation of the T6SS mutant were comparable to wild-type levels in a standard chitin transformation assay using DNA only (Supplementary Fig. 3). Transformation efficiency of the Δ*hcp1*,*2* double mutant was restored to wild-type levels when co-incubated with protozoa (Fig. 4C, D). As with naked DNA, transformation of the suicide plasmid pCVD442::Δ*rtx* provided within *E. coli* WM3064 was not enhanced in the presence of protists and gave similar efficiencies to pKC01 in the absence of protozoa (Fig. 4C, D).

### The *V. cholerae* SOS response is required for *intIA* transcription and gene cassette transformation within protozoa

The integron-integrase (*intIA*) gene is regulated by the SOS response, a global response to DNA damage that involves interaction of the RecA recombinational repair protein with the repressor protein, LexA [13, 15]. To determine whether *intIA* is induced through the SOS response in protozoal phagosomes, a *recA* mutation was constructed in the integron-integrase reporter strain. The mutant was co-incubated with *T. pyriformis* or *A. castellanii* and analyzed by CLSM and spectrofluorometry. Confocal microscopy revealed no GFP fluorescence in the *recA* mutant background in *T. pyriformis*, (Fig. 1A), a finding supported by spectrofluorometry (Fig. 2A). In *A. castellanii*, wide-field fluorescence microscopy revealed low levels of GFP fluorescence (Fig. 1B) that were substantially reduced compared to the *recA*^+^ strain with a tenfold reduction as quantified by spectrofluorometry (Fig. 2B). RT-qPCR of the *intIA* transcript showed a 27.6-fold reduction in the *recA* mutant compared to the wild type in *T. pyriformis* (Fig. 3A). Transcription of *intIA* was higher in *A. castellanii* compared to *T. pyriformis*, although it was 2.6-fold lower than the wild type indicating non-SOS transcription of *intIA* (Fig. 3B), although this level was insufficient for production of transformants in the gene cassette transformation assay (Fig. 4). The transformation assay revealed non-detectable transformation of pKC01 (either as free DNA or within *E. coli* WM3064) in the *recA* mutant strain in both protozoal treatments and no protozoa controls (Fig. 4). Complementation restored the *recA* mutant to wild type levels in all assays (Figs. 2–4). Transformation of DNA into *V. cholerae* is single-stranded therefore, pKC01 must be first circularized by RecA before integron-integrase mediation recombination can occur [27]. To confirm that the lack of pKC01 transformants in the *recA* mutant was due to a dysfunctional SOS-response rather than the requirement for RecA in circularizing pKC01, a *lexA*(ind^-^) mutant was engineered that produces a LexA variant resistant to RecA cleavage preventing SOS-induction but maintaining RecA activity [13]. The *lexA*(ind^-^) gave non-detectable transformation of pKC01 (either as free DNA or within *E. coli* WM3064) confirming that the SOS-response is required for transformation in the presence of protozoa.

### Oxidative radicals produced by protozoa are required for SOS induction of *intIA* and LGT in *V. cholerae*

**Fig. 5:**
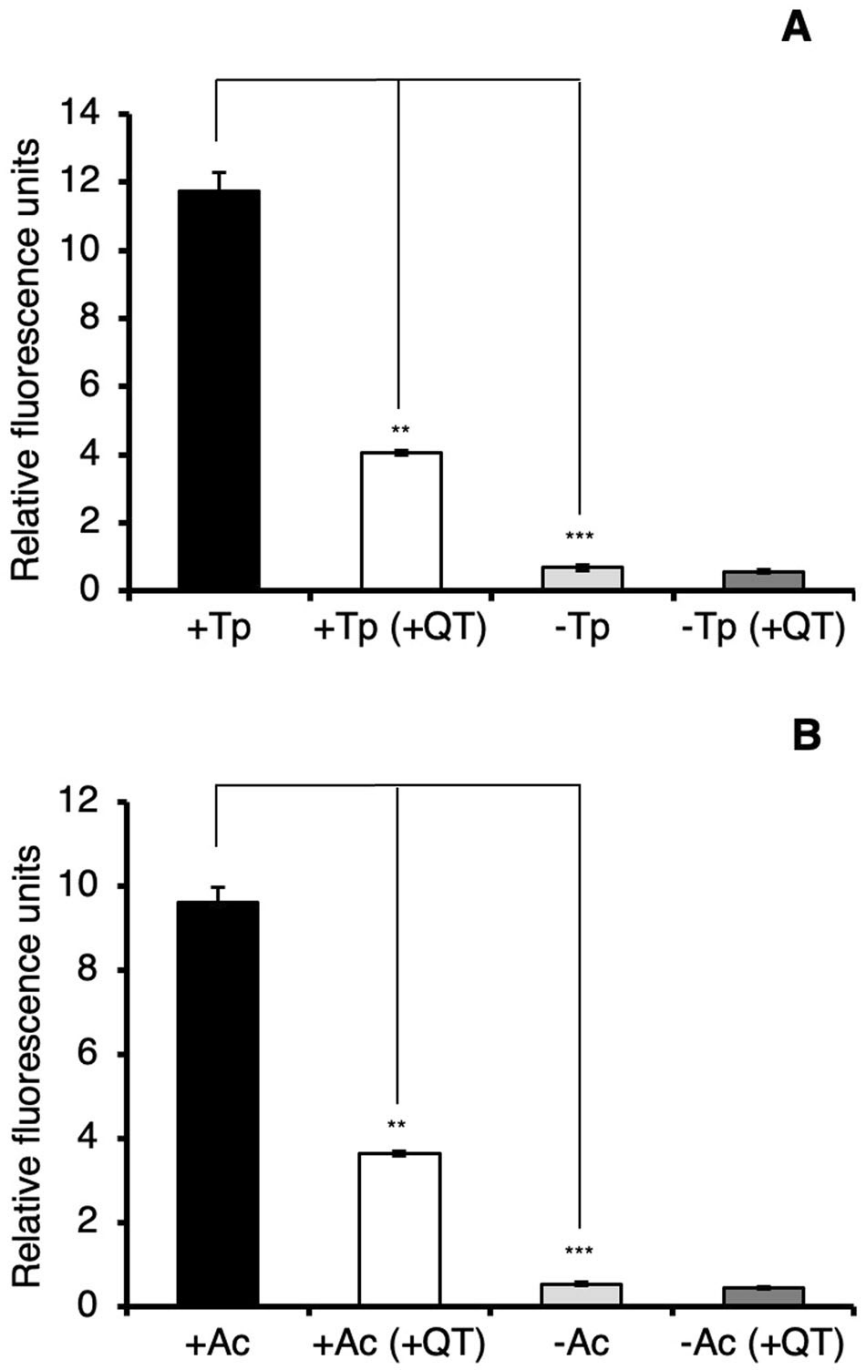
Oxidative radical production inside protozoa. Following co-incubation with wild-type (WT) *V. cholerae* A1552, the level of internal oxidative radicals in *T. pyriformis* (**A**) and *A. castellanii* (**B**) were measured using the fluorescent dye DCF-DA and fluorescence spectrometry. +QT indicates the addition of 100 mM of the quencher, thiourea, to the co-incubation. Error bars represent the standard deviations of three independent experiments. Significance was calculated using one-way ANOVA and Tukey’s multiple comparisons test (**p < 0.001; ***p < 0.0001).

Protozoa introduce oxidative radicals, such as reactive oxygen species, in phagosomes following internalization of bacteria. We therefore hypothesized that oxidative radicals may be inducing the bacterial SOS response leading to *intIA* transcription and subsequent pKC01 integration. First, we quantified the level of oxidative radical production in *T. pyriformis* and *A. castellanii* during digestion of wild-type *V. cholerae* with and without treatment with thiourea to confirm that thiourea reduces oxidative radical activity. After 4 h of feeding, protozoal cells were purified and lysed with 1% Triton-X to release intracellular oxidative radicals, which were then stained with the reporter dye, 2′,7′-Dichlorofluorescin Diacetate (DC-FDA). Spectrofluorometry showed that the addition of thiourea reduced intracellular oxidative radicals by ~3-fold in both *T. pyriformis* and *A. castellanii* (Fig. 5). Although some DCF-DA fluorescence was observed in the controls, it was 17- and 18-fold higher in the presence of *T. pyriformis* and *A. castellanii*, respectively.

Using CLSM we showed that the addition of thiourea resulted in a substantial reduction in GFP-fluorescing cells in both protozoa (Fig. 1). Spectrofluorometry showed that the addition of thiourea resulted in a 7.4- and 2.3-fold reduction of GFP fluorescence in *T. pyriformis* and *A. castellanii*, respectively, compared to the no thiourea controls (Fig. 2). RT-qPCR showed thiourea treatment resulted in a 1.8- and 1.6-fold decrease in *intIA* transcription in the wild type compared to the no thiourea *T. pyriformis* and *A. castellanii* controls respectively (Fig. 3). Additionally, thiourea decreased pKC01 transformation in the chitin-grown wild type by 535-fold and 47-fold in *T. pyriformis* and *A. castellanii*, respectively (Fig. 4). Interestingly, although transformation of free pKC01 was detected in the no protozoa controls (Fig. 4), thiourea had no impact on transformation levels indicating that increased levels of pKC01 transformation in protozoa is due solely to the production of oxidative radicals. These oxidative radicals induced the SOS response above levels occurring during LGT alone. When pKC01 was provided by *E. coli* WM3064, thiourea was found to reduce transformation frequency in the wild type by 389- and 45-fold in *T. pyriformis* and *A. castellanii*, respectively (Fig. 4). Complemented mutants incubated with protozoa in the presence of thiourea showed transformation efficiency similar to the wild type (Fig. 4).

## Discussion

**Fig. 6:**
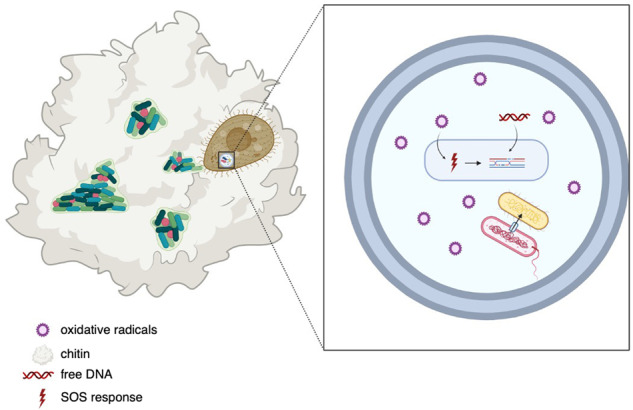
Illustration describing enhanced transformation in *V. cholerae* inside the protozoal food vacuole. *V. cholerae* forms mixed biofilms on chitin substates in the water column becoming naturally competent. Protozoal feeding results in mixed bacteria in the food vacuole where oxidative radicals induce the *V. cholerae* SOS response increasing expression of the integron-integrase and enhancing integration of transferred gene cassettes. *V. cholerae* utilizes type VI secretion to acquire DNA from other bacteria in the food vacuole.

*V. cholerae* is a highly infective human pathogen but most of its lifecycle is spent in the aquatic environment as biofilms on chitinous surfaces [28, 29] where it becomes naturally competent and therefore, capable of LGT-mediated genetic diversification [6]. In the environment, *V. cholerae* is present in mixed communities in the water column and in biofilms [2] and is under constant grazing presure from protozoa [30]. Although protozoa may have feeding preferences, they will graze upon different bacterial groups [31–33] accumulating and concentrating diverse bacteria in food vacuoles [3], providing an opportunity for genetic exchange [3, 34]. In this study, we demonstrate the uptake of *V. cholerae* and *E. coli* by two different predators, the filter feeding *T. pyriformis* and the raptorial feeder, *A castellanii*. We demonstrate that gene cassette transformation is enhanced in *V. cholerae* when co-incubated with protozoa, and that this is due to increased expression of the integron-integrase and gene cassette integration in the phagosome. While other studies have demonstrated that food vacuoles are micro-niches for conjugation and transduction [34–37], we identify that the conditions of the food vacuole enhance transformation by upregulating the integrative step of LGT. We show that *V. cholerae* was transformed with a gene cassette from *E. coli* when they were inside the food vacuoles of both *T. pyriformis* and *A. castellanii*, and that this required a functional T6SS indicating that it was utilized to lyse neighboring cells to access DNA. That we detected no gene cassette transformants in the controls lacking protozoa indicates that protozoa are important in facilitating the close association of donor and recipient cells required for T6SS-mediated LGT (see Fig. 6 for an illustrative demonstration). Additionally, we observed that transformation rates differed between our two protozoal models which is likely related to feeding rates being higher in the *T. pyriformis* ciliate versus the surface-feeding *A. castellanii* [38] which affects the number of bacteria exposed to the phagosome environment.

Another important aspect of this study was demonstrating that the integron-integrase is induced through the SOS response via the action of protozoan-produced oxidative radicals in the phagosomes. Laterally transferred DNA activates the SOS reponse which induces the integron-integrase thus linking DNA transfer with DNA integration [13, 18]. However, in this study, it is the presence of oxidative radicals that are shown to enhance integron-integrase mediated gene cassette transformation in *V. cholerae*. Conversely, RecA-mediated homologous recombination was not enhanced in the presence of protists indicating that at least in this context, there is a limit to *recA* induction and confirming that increased transformation of pKC01 is due to upregulated integron-integrase and not increased DNA uptake. Since thiourea reduced transformation efficiency in co-incubation with protozoa but not when *V. cholerae* was grown on chitin, it can be concluded that SOS is activated above the level induced by the presence of ssDNA from LGT and is due to oxidative radicals. Additionally, low levels of non-SOS regulated integron-integrase induction was observed in *A. castellanii* although this was insufficient to produce transformants. Nevertheless, it shows that SOS-independent integron-integrase induction occurs in the phagosome with catabolite control a likely pathway since it has previously been observed to control the *V. cholerae intIA* [39]. As the SOS response is conserved in all bacteria and is important in generating genetic diversity through mutation, recombination and LGT [14], protozoal predation is likely an important driver of bacterial adaptation. Importantly, the class 1 integron-integrase which is important in the lateral transfer of antibiotic resistance genes is also regulated by the SOS response [13] and so the protozoal phagosome environment may be an important niche for transfer of antibiotic resistance genes in other bacteria. Additionally, prior studies have reported that the SOS response enhances the transfer of multiple MGEs in bacteria including the lysogenic CTXϕ in *V. cholerae* [40] and others involved in antibiotic resistance [13, 18, 41–46] so, lateral transfer of DNA in this context likely goes beyond gene cassettes.

As a defense against grazing, generating genetic diversity is important for producing strain(s) that may have an advantage. Apart from the acquisition of new gene cassettes, induction of the integron-integrase causes variation in the cassette array such as rearrangements (observed in this study with relocation of a cassette into *attI* in one of our transformants) or deletions of which the latter is known to cause changes in surface properties and biofilm formation in at least one *Vibrio* species [47, 48]. Additionally, the SOS response can cause other genetic changes such as elevated mutation, recombination or movement of MGEs [14]. Due to the overlap of protozoal survival factors with virulence factors, it is hypothesized that protozoal predators may contribute to the emergence of pathogens [49] and our study demonstrating a link between generation of genetic diversity and protozoal grazing selection supports this idea. As *V. cholerae* is released from protozoa into the environment as expelled food vacuoles (EFVs) and these packaged cells are more resistant to environmental stress and more infectious in vivo [5], the cycling of *V. cholerae* through protozoa in EFVs provides opportunity for creation of genetic variants that survive long enough to be “tested” in human disease. Taken together, this study shows that the food vacuoles of protozoa may be an important niche where *V. cholerae* generates genetic diversity through SOS-induced LGT.

## Supplementary information

Supplementary Material

Supplementary Video 1
