# Supplementary material for: Protozoal food vacuoles enhance transformation in Vibrio cholerae through SOS-regulated DNA integration
Source: ISME J. 2022 May 16;16(8):1993–2001. doi: 10.1038/s41396-022-01249-0 (PMC9296650; doi:10.1038/s41396-022-01249-0)
Supplement: Supplementary file 1 — Supplementary Material [file 41396_2022_1249_MOESM1_ESM.docx]

**Supplementary Information**

**Protozoal food vacuoles enhance transformation in *Vibrio cholerae* through SOS-regulated DNA integration**

Md Hafizur Rahman^1^, Khandaker Rayhan Mahbub^1,3^, Gustavo Espinoza-Vergara^2^, Angus Ritchie^1^, MD Mozammel Hoque^2^, Parisa Noorian^2^, Louise Cole^2^, Diane McDougald^2^, Maurizio Labbate^1,*^

^1^School of Life Sciences, Faculty of Science, University of Technology Sydney, Sydney, New South Wales, Australia

^2^Australian Institute for Microbiology & Infection, University of Technology Sydney, Sydney, New South Wales, Australia.

^3^South Australian Research and Development Institute, Urrbrae, South Australia, Australia

^*^Corresponding: [maurizio.labbate@gmail.com](mailto:maurizio.labbate@gmail.com)

**Supplementary Table 1.** Strains and plasmids used in this study

| **Strain or plasmid** | **Relevant genotype and relevant phenotype** | **Reference or source** |
| --- | --- | --- |
| **Bacterial strains** | | |
| ***V. cholerae*** |  |  |
| A1552 | Wild type, O1 EI Tor, smooth variant | (1) |
| A1552 *intIA*::*gfp* | A1552, contains *gfp* in transcriptional fusion with *intIA;* Cm^R^, Kan^R^ | This study |
| A1552 *intIA*::*gfp,* Δ*recA* | A1552 *intIA*::*gfp*, contains a deletion of *recA* interrupted with a Spc^R^ gene; Cm^R^, Kan^R^ Spc^R^ | This study |
| A1552 Δ*intIA* | A1552, contains an in frame deletion of *intIA* | This study |
| A1552 Δ*recA* | A1552, contains a deletion of *recA* interrupted with a Spc^R^ gene; Spc^R^ | This study |
| A1552 *lexA*(ind^-^) | A1552, containing in an A91D allele in *lexA* | This study |
| A1552 Δ*hcp1*,*2* | Deletion of alleles VC1415 and VCA0017 encoding Hcp that polymerise to form the nanotube of the type VI secretion system | (2) |
| C6706_TnVCH35 | *V. cholerae* C6706, O1 EI Tor, smooth variant with TnFGL3 inserted into a gene cassette (locus tag RS15635 in *V. cholerae* N16961, NZ_CP028828) | (3) |
| ***E. coli*** |  |  |
| WM3064 | *thrB*1004 *pro* *thi* *rpsL* *hsdS* *lacZ*ΔM15 RP4-1360 Δ(*araBAD*)567 Δ*dapA*1341::[*erm pir*] | (4) |
| ∏3813 | *lacI*^q^ *thi-1 supE44* *endA1 recA1 hsdR17* *gyrA462* *zei-298*::Tn*10* Δ*thyA*::(*erm–pir-116*), Tc^R^, Em^R^; Resistant to the activity of CcdB. | (5) |
| **Plasmids** | | |
| p4640 | *gfp* in transcriptional fusion with *intIA*, Cm^R^, Kan^R^ | (6) |
| pKC01 | Derived from TnFGL3 transposon. Artificial gene cassette and plasmid with a conditional oriR6K; Kan^R^ | This study |
| pKD3 | Source of chloramphenicol resistance gene flanked by FRT sites; Cm^R^ | (7) |
| pOriVn_700_ | Source of spectinomycin resistance gene; Spc^R^ | (8) |
| pSU-pBAD | Arabinose expression vector. Used for complementation experiments; Cm^R^ | (5) |
| pSU-pBAD::*intIA* | pSU-pBAD with *intIA* from *V. cholerae* in front of the arabinose-inducible promoter | This study |
| pSU-pBAD::*recA* | pSU-pBAD with *recA* from *V. cholerae* in front of the arabinose-inducible promoter | This study |
| pSU-pBAD::*hcp1* | pSU-pBAD with *hcp1* from *V. cholerae* in front of the arabinose-inducible promoter | This study |
| pSW4426T | Suicide vector containing an *araC*-P_BAD_-*ccdB* cassette counter-selectable marker; Cm^R^, Sp^R^ | (5) |
| pSW4426T::*lexA* | pSW4426T with *lexA* from *V. cholerae* | This study |
| pSW4426T::*lexA*(ind-) | pSW4426T with *lexA*(ind^-^) from *V. cholerae* | This study |
| pCVD442::Δ*rtx* | pCVD442 with *rtx* from *V. cholerae* interrupted with a chloramphenicol marker, Cm^R^ | (9) |
| **Protozoan strains** | | |
| *T. pyriformis* | Wild type | ATCC 205063 |
| *A. castellanii* | Wild type | ATCC 30234 |

Cm^R^, chloramphenicol resistant, Spc^R^, spectinomycin resistant; Kan^R^, kanamycin resistant, Tc^R^, tetracycline resistant, Em^R^, erythromycin resistant

**Supplementary Table 2.** Primers used in this study

| Primer | Sequence (5`-3`) | Target | Reference |
| --- | --- | --- | --- |
| ompW_F | CACCAAGAAGGTGACTTTATTGTG | Species-specific *ompW* in *V. cholerae* | (10) |
| ompW_R | GGTTTGTCGAATTAGCTTCACC |  |  |
| kan_F | ATT CAA CGG GAA ACG TCT TG | Kanamycin resistance gene (*nptII*) | This study |
| kan_R | CGA GCA TCA AAT GAA ACT GC |  |  |
| gyrA_F | CACGAACTCTTGGCAGACCT | *gyrA* in *V. cholerae* | (11) |
| gyrA_R | CAATACCAGATGCGCCGTTG |  |  |
| intIA-1F | TCAAGCGCATACACTCCATC | *intIA* in *V. cholerae* | This study |
| intIA-1R | TTCAACGCTCGCAACTAGAA |  |  |
| IntIA-F2 | CGCGAACACTTAACAAAAACTGG | 5’ end of the integron-integrase of *V. cholerae* and 1^st^ gene cassette in array - amplifies region spanning *attI* | This study |
| VCH3-R | TCGTGAACACCGAAAAGACA |  |  |
| VCH26-F | GCAGCTATGGGATGTCGCAA | 5’ end of the 26^th^ gene cassette relative to *attI* in the *V. cholerae* A1552 integron | This study |
| VCH38-R | GCATGCCTGCTCATATAGCCT | 3’ end of 36^th^ gene cassette relative to *attI* in the *V. cholerae* A1552 integron | This study |
| recA-F | ATTGAAGGCGAAATGGGCGATAG | *recA* in *V. cholerae* | (12) |
| recA-R | TACACATACAGTTGGATTGCTTGAGG |  |  |
| intIA_up_F | ATAATGCCTCGCGTAAAACG | *intIA* upstream region  Underlined region overlaps with cat_F | This study |
| intIA_up_R | TCCAGCCTACACTGGGGGCTGATATACGTACC |  |  |
| intIA-down_F | GGAGGATATTCATATGTTGCATAAATTCGCGAACAC | *intIA* downstream region  Underlined region overlaps with cat_R | This study |
| intIA_down_R | CCCATTGCGTTAAATACCAT | First gene cassette inserted at *attI* in A1552 |  |
| cat_F | GTGTAGGCTGGAGCTGCTTC | FRT-*cat*-FRT cassette from pKD3 | (7) |
| cat_R | CATATGAATATCCTCCTTAG |  |  |
| recA-up-F | TTCAGGCGGTCGAGCATAAT | Upstream region of *recA* in *V. cholerae*  Underlined region overlaps with aadA7-F | This study |
| recA-up-R | AGATCGTTTTGCATGATGGAGCCTTTACCA |  |  |
| recA-down-F | GGGCGAATTGCTGATCGCTGAAACATCTTCTG | Downstream region of *recA* in *V. cholerae*  Underlined region overlaps with aadA7-R | This study |
| recA-down-R | GAAGCCGTAGGTGTCGTAGAGT |  |  |
| aadA7-F | TCCATCATGCAAAACGATCTCAAGAAGATC | *aadA7* from pOriVn_700_ | This study |
| aadA7-R | CAGCGATCAGCAATTCGCCCTATAGTGAGT |  |  |
| pSU-pBAD-F | AGTCTAGACAGCGCTTTTCC | Amplification of the pSU-pBAD backbone |  |
| pSU-pBAD-R | GTTTCACTCCATCCAAAAAAAC |  |  |
| Cloning intIA_F | TTTTGGATGGAGTGAAACGCTAAAGACGGGATAATGGGCTTA | Amplification for *intIA* for Gibson cloning into pSU-pBAD  Underlined region overlaps with pSU-pBAD | This study |
| Cloning intIA_R | AAAAGCGCTGTCTAGACTATGAAATCCCAGTTTTTGTTAAGTG |  |  |
| Cloning recA_F | TTTTGGATGGAGTGAAACGCGAGAATAAACAGAAGGCACTTG | Amplification for *intIA* for Gibson cloning into pSU-pBAD  Underlined region overlaps with pSU-pBAD | This study |
| Cloning recA_R | AAAAGCGCTGTCTAGACTAGCCTGCCGATTAAAACTCTTC |  |  |
| Cloning hcp 1_F | TTTTGGATGGAGTGAAACGATGCCAACTCCATGTTATATCTC | Amplification for *hcp1* for Gibson cloning into pSU-pBAD  Underlined region overlaps with pSU-pBAD | This study |
| Cloning hcp 1_R | AAAAGCGCTGTCTAGACTTTACGCTTCGATTGGCTTACG |  |  |
| pSW4426T-F | ACTAGTTCTAGAAAAAAAAAGCC | Amplification of pSW4426T backbone | This study |
| pSW4426T_R | GATATCAAGCTTATCGATACCG |  |  |
| LexA-F | GTATCGATAAGCTTGATATCTCAGCAAGTCGACGTTGG | Amplification of *lexA* for Gibson cloning into pSW4426T  Underlined region overlaps with pSW4426T | This study |
| LexA-R | TTTTTTTTTCTAGAACTAGTGGCCTGCACCGACATTAATG |  |  |
| LexA-Ind-F | CGTTGCC**GAT**GGTGAACCGATTCTT | Amplification of pSW4426T::*lexA* for incorporating the A91D allele and creating  pSW4426T::*lexA*(Ind^-^)  Bold nucleotides indicate modified codon in *lexA* | This study |
| LexA-Ind-R | GTTCACC**ATC**GGCAACGCGGCCAAT |  |  |
| LexA-F-ARMS | CAACCCGCGCCGAAATTGCT | Used in ARMS PCR to differentiate between the *lexA* and *lexA*(Ind^-^) alleles | This study |
| LexA-R-ARMS | CTTGCGGGCGGAACATGCTC |  |  |
| WT_LexA-F-ARMS | ATTGGCCGCGTTGCCGCG |  |  |
| Ind_LexA-R-ARMS | ATGCTCTTGAGCAAGAATCGGTTCACCAT |  |  |

**
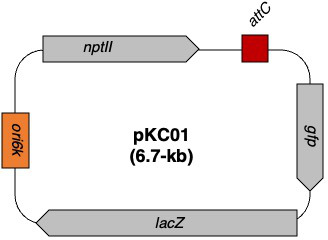
**

**Supplementary Figure 1. Schematic of pKC01.** pKC01 contains an *ori6k* permitting replication in λ-pir strains, a kanamycin resistance gene (*nptII*), *gfp* and *lacZ*. Not drawn to scale.

**
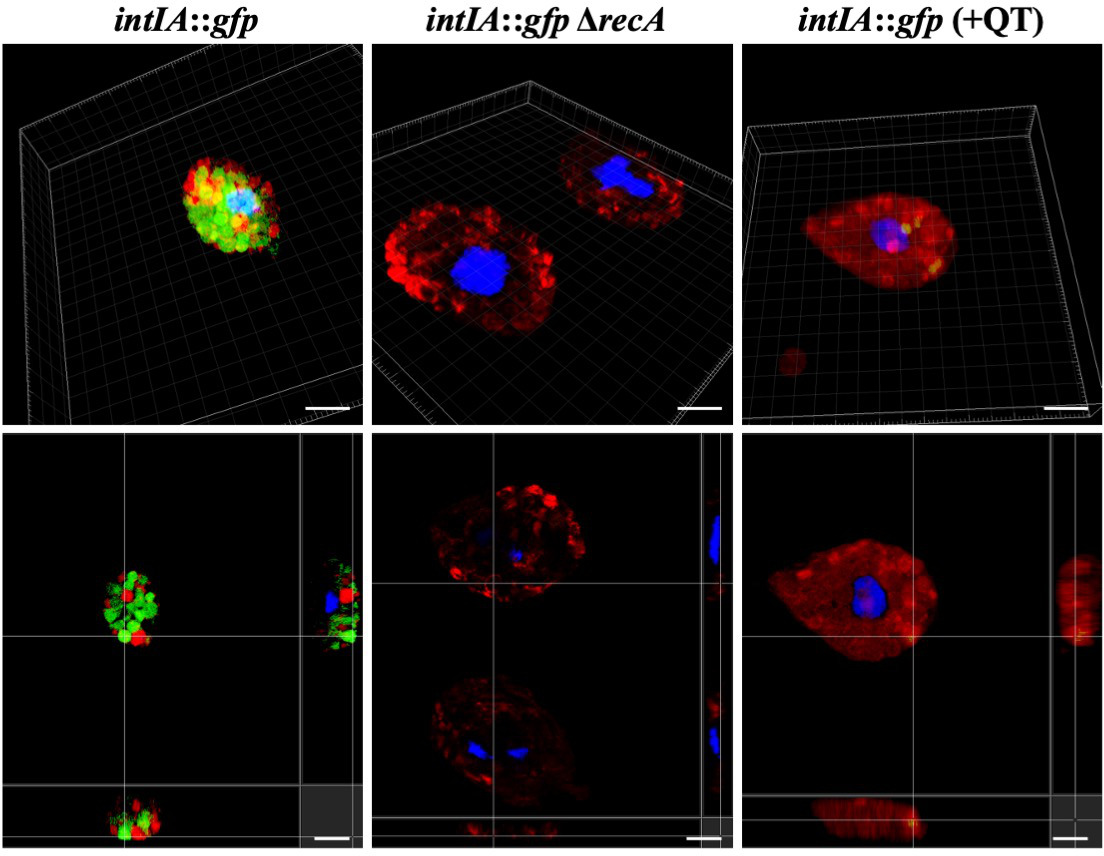
**

**Supplementary Figure 2. Microscopy of *T. pyriformis* after co-culture with *V. cholerae.*** The *V. cholerae* integron-integrase reporter strain (*intIA*::*gfp*) contains a transcriptional fusion of *intIA* and *gfp.* The *intIA*::*gfp,*Δ*recA* strain is the reporter strain lacking a functional *recA*. Samples were stained with DAPI and FM4-64FX with images showing signals from GFP (green), DAPI fluorescence (blue) and FM4-64FX (red) fluorescence. A. Top panels show 3D composite images processed using Imaris 3D-visualization software to display a volume view. Lower panels show 3-D orthogonal views of the same samples to show XY, XZ and YZ views in each case. +QT indicates the addition of 100 mM of the quencher, thiourea. Scale bar 10 µM. Note that food vacuoles containing fluorescing *intIA::gfp*  reporter cells are observed throughout the protozoal cell. Fluorescing cells are absent or significantly reduced in the *intIA*::*gfp,*Δ*recA* strain and *intIA*::*gfp* strain treated with thiourea.

**
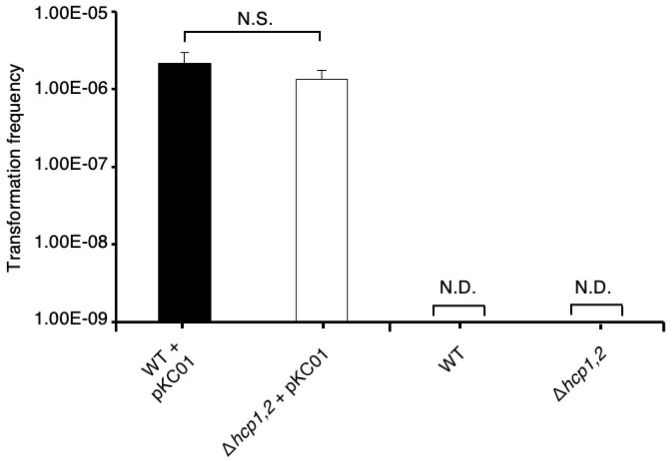
**

**Supplementary Figure 3**. Frequency of gene cassette integration in *V. cholerae* strains grown on chitin. No significant (N.S.) (p<0.005) difference in transformation frequency was found between the WT and Δ*hcp1,2* strain unable to make T6SS using 1 µg of pKC01. N.D. indicates non-detectable transformants. Error bars represent the standard deviations of three independent experiments.

**References**

1. Yildiz FH, Liu XS, Heydorn A, Schoolnik GK. Molecular analysis of rugosity in a *Vibrio cholerae* O1 El Tor phase variant. Mol Microbiol. 2004;53(2):497-515.

2. Ishikawa T, Rompikuntal PK, Lindmark B, Milton DL, Wai SN. Quorum sensing regulation of the two *hcp* alleles in *Vibrio cholerae* O1 strains. PLoS One. 2009;4(8):e6734.

3. Cameron DE, Urbach JM, Mekalanos JJ. A defined transposon mutant library and its use in identifying motility genes in *Vibrio cholerae*. Proc Natl Acad Sci USA. 2008;105(25):8736-41.

4. Saltikov CW, Newman DK. Genetic identification of a respiratory arsenate reductase. Proc Natl Acad Sci. 2003;100(19):10983-8.

5. Le Roux F, Binesse J, Saulnier D, Mazel D. Construction of a *Vibrio splendidus* mutant lacking the metalloprotease gene *vsm* by use of a novel counterselectable suicide vector. Appl Environ Microbiol. 2007;73(3):777-84.

6. Baharoglu Z, Bikard D, Mazel D. Conjugative DNA transfer induces the bacterial SOS response and promotes antibiotic resistance development through integron activation. PLoS Genet. 2010;6(10):e1001165.

7. Datsenko KA, Wanner BL. One-step inactivation of chromosomal genes in *Escherichia coli* K-12 using PCR products. Proc Natl Acad Sci. 2000;97(12):6640-5.

8. Le Roux F, Davis BM, Waldor MK. Conserved small RNAs govern replication and incompatibility of a diverse new plasmid family from marine bacteria. Nucleic Acids Res. 2011;39(3):1004-13.

9. Espinoza-Vergara G, Noorian P, Silva-Valenzuela CA, Raymond BBA, Allen C, Hoque MM, et al. *Vibrio cholerae* residing in food vacuoles expelled by protozoa are more infectious in vivo. Nat Microbiol. 2019;4(12):2466-74.

10. Nandi B, Nandy Ranjan K, Mukhopadhyay S, Nair GB, Shimada T, Ghose Asoke C. Rapid method for species-specific identification of *Vibrio cholerae* using primers targeted to the gene of outer membrane protein OmpW. J Clin Microbiol. 2000;38(11):4145-51.

11. Noorian P, Hu J, Chen Z, Kjelleberg S, Wilkins MR, Sun S, et al. Pyomelanin produced by *Vibrio cholerae* confers resistance to predation by *Acanthamoeba castellanii*. FEMS Microbiol Ecol. 2017;93(12):fix147.

12. Amin Marashi SM, Rajabnia R, Imani Fooladi AA, Hojati Z, Moghim S, Nasr Esfahani B. Determination of *ctxAB* expression in *Vibrio cholerae* Classical and El Tor strains using real-Time PCR. Int J Mol Cell Med. 2013;2(1):9-13.

**DNA sequence of transformant with relocated gene cassette**

>Transformant 4

CGCGAACACTTAACAAAAACTGGGATTTCATATAGTTCTCACTGAATATTTAACTGGTTATTTGTACAGTATTTGTTGGTTGTTTTTATGTCAAGAGGCTATACAGACATCAGCAATCTATAAGCTGAGATTTTTGAATGGTGTGATGCTCAACATACTGATTTAGAAGGTTGTTATGGTAGTATGCACCCAGTGGTCTATTAATTAGATAGCGGTAGCCTACCTGTTGGAAAGGTAAGAAGCTGTCTAGAAAGCGTTAGCCATTTGGGAGAACTATGGAAAATTTAACAAATGATGTAATTACAATTTTACAGTATCTGCTCCCGGGGTTTGTGTCTGCTTGGGTGTTTTATAGTTTGACCTCTTATCCCAAGCCATCACAATTTGAGCGTGTGGTACAGGCTCTGATTTTTACTATTTTTATACAAGCAATTGTTGTGGTGGTTGAGTTCTCAGTCCGTTGGTTGTGTGCTACGTATGAAATTACAGGAGTACCGGACAAGTTTGGGTTAGTTGGTTCTGTTGTGACAGCTATCTTACTTGGGGTTGCATTTGCAGGCTTTGCCAACAATGACCTATTGCATAAGTGTCTAAGAAGGTGTTTGATCACAAGAGAAACATCATATCCTTCGGAATGGTTTGGTGCCTTTTTACAGGACGTTACTTTTGTTGTCTTACACCTAAATGATGAACGAAGGTTATATGGCTGGCCAATTGAATGGCCTTCAGAACCACATAAAGGTCATTTTGTTATCGCAGACCCGTCTTGGCTCAACGATGATGGCACTGAGGCAGAAATAACAGGTGTATCTAAACTTGTTATTAGCGCTAGTGAAGTAAAATGGGTGGAATTTCTAGAGAAAAACTGGGAGTAGTTATATGTCAAAAAAAGGTTCAAACCCTCCACCACCAAGTGGAAGAACTGGTTGGACTACAGATGGTGCAAATCCAGCTAGAACACCTCAGAACGTAAGGCCTAGCCCACCACCTCCGCCGCCACCGAAAAAAAATGGCTAACAAAGCATTTAAGAGTGATTCCCAACGCTTGGCATTTTCAGTGCTAATCGTTGGGTTTTGTGTTTATGGTGGCATGGTTGAGTCTCGTGTTAGCGTTGCTCACACCTTAATGCGGCGTTAGGCTAATCAGGGAGATAAAGATGTCACTTTGGGTTTTGGTTCCGCTTTCATTTTTCCAGCTTGGAGTAGGTAGCATTATTGGTTTTGGGCTTATATTTCTCTCAGGTATAGATGCTTTCTGAGTTCAACAATAATGTTTGTGTAGCGCTATGGTTTCTATATGTCTTTTCGGTGTTCACGA
